# Randomized controlled trial on **Pri**ngle **Ma**neuver to reduce blood **l**oss during **st**apler **hep**atectomy - **PriMal StHep**

**DOI:** 10.1186/s12893-019-0524-6

**Published:** 2019-06-10

**Authors:** Philipp Houben, Ulf Hinz, Phillip Knebel, Markus K. Diener, Arianeb Mehrabi, Peter Schemmer

**Affiliations:** 1 Department of General, Visceral and Transplant Surgery, University of Heidelberg, Im Neuenheimer Feld 110, 69120 Heidelberg, Germany; 2 Current address: Division of Transplant Surgery, Department of Surgery, Medical University of Graz, Graz, Austria

**Keywords:** Liver resection, Pringle maneuver, Blood loss

## Abstract

**Background:**

Extended liver resections still bear the risk of severe haemorrhage. Moreover, the amount of blood loss during liver resection determines the need for perioperative blood transfusions and is of prognostic relevance in oncologic surgery. Even though there is an ongoing debate about its effectiveness and tolerable duration, the Pringle Maneuver (PM) as an occlusion of the hepatic inflow is routinely applied to reduce blood loss during parenchymal dissection. In combination with the stapler resection technique, PM is expected to minimize blood loss during major liver resection safely due to the short parenchymal dissection duration.

**Methods:**

In a single center prospective, randomized, controlled, parallel, confirmatory trial the combination of PM and stapler resection technique in patients undergoing right and left hepatectomies will be tested against the control group that applies stapler resection without the use of PM. The primary endpoint of the study is the total intraoperative blood loss. The measurement of the intraoperative blood loss is conducted with respect to all handled rinse fluids during surgery and by weighing used swabs to generate accurate and comparable data. Secondary endpoints include intra- and postoperative blood transfusion requirements, liver function parameters and the 90-day mortality rate. A sample size of fifty-three patients in either group was calculated to detect a clinically significant difference in blood loss of at least 450 ml with an α of 5% at 80% power. The individual follow-up will be 90 days.

**Discussion:**

This is the first clinical trial to test the combination of PM and stapler resection technique as a means to reduce intraoperative blood loss in hepatic left or right resection. Given the short parenchymal dissection duration in stapler resection, PM is expected to be applied shortly without compromising liver function postoperatively.

**Trial registration:**

The PriMaL StHep trial has been prospectively registered to the German Clinical Trial Registry (WHO ID: DRKS00010427) on April 21st. 2016.

## Background

Resection is the standard treatment for a variety of benign and malignant pathologies of the liver. Depending on the size of the finding and the nature of the disease, resections can be performed as atypical or anatomical resections. Major liver resections, including left and right hepatectomies and their extensions listed in the Brisbane 2000 terminology [1], still carry the risk of severe hemorrhage [2]. Perioperative blood loss (BL) not only affects short-term outcome after surgery, it has been shown that extensive blood loss and transfusion of blood products are related to increased tumor recurrence in Hepatocellular Carcinoma (HCC) patients [3–6]. Voogt, et al. reported the same finding in colorectal cancer patients [7]. Accordingly, perioperative blood transfusions in colorectal metastases resection were found to be associated with impaired perioperative outcome, increased recurrence rate, and reduced long term survival [8, 9]. Currently, it was shown that a reduction of blood loss of 400 ml was associated with the absence of blood transfusions in a series of 186 liver resections [10]. Limiting intraoperative BL to avoid related blood transfusion therefore remains mandatory in liver surgery for malignancies; measures include resection along anatomical planes, maintenance of low central venous pressure (CVP), and blood saving resection techniques. Due to the varying application of these measures and the presence of other potential confounders, such as selection and reporting bias and uncontrolled circumstantial effects, case series and uncontrolled studies can only reveal limited understanding of strategies to successfully reduce the blood loss in major liver resections. Therefore, this single center Randomized Controlled Trial (RCT) which applies a highly standardized surgical technique that is limited to the clearly defined surgical procedure of left or right hepatectomy, was designed to evaluate this matter.

Conventional resection methods such as the Finger Fracture and the Clamp Crush techniques are challenged by the use of several innovative parenchymal dissection devices. Interestingly, a Cochrane database review of innovative and conventional liver resection techniques from 2009 revealed the traditional Clamp Crush technique to provide comparable or lower BL at lowest cost [11]. Traditionally, different modes of vascular occlusion are applied to further limit blood loss in liver resection. Total Hepatic Vascular Exclusion (THVE) includes the temporary ligation or clamping of the portal triad with simultaneous occlusion of the hepatic veins to prevent retrograde hemorrhage during resection, whereas the classic Pringle Maneuver (PM) only applies occlusion of the arterial and portal inflow. Due to the relative ischemia tolerance of the liver, Intermittent Pringle Maneuver (IPM) is utilized to facilitate extended resections with limited BL. There is little evidence from well-designed clinical trials to justify the widespread use of these techniques. A meta-analysis from 2008 revealed no advantage for the use of portal triad clamping in terms of perioperative outcome [12]. Additionally, the innovation of surgical techniques has led to a reduction in intraoperative BL, making inflow control dispensable. Through the use of vascular staplers, the duration of parenchymal resection has been significantly reduced to less than ten minutes for major hepatic resections with excellent safety [13, 14]. Nevertheless, a relevant blood loss still occurs, even in anatomic resections. This might be attributed to bleeding from the hepatic venous system, uncontrolled portoportal contralateral collaterals and failing to follow the precise anatomical segemental border during parenchyma dissection. Combining vascular occlusion techniques and stapler resection could help to further minimize intraoperative BL. It is assumed that the short duration of portal triad clamping has no negative impact on the perioperative outcome when applied in stapler hepatectomy. Therefore, this RCT was designed to evaluate the potential reduction of BL by the combination of stapler hepatectomy and PM.

### Objective

The trial’s objective is to test if PM reduces the intraoperative BL during left or right stapler hepatectomy. Furthermore, perioperative outcome is analyzed to evaluate the safety of the combination of PM and stapler resection technique.

### Hypotheses

Null hypothesis: The intraoperative BL in both groups does not differ to a clinically relevant extent with (H0): BL 1 = BL 2.

Alternative hypothesis: The intraoperative BL in both groups differs to a clinically relevant extent with (H1): BL 1 ≠ BL 2.

### Formal trial design

Single center prospective, randomized, controlled, parallel, open, confirmatory trial.

### Primary endpoint

- Total intraoperative BL during stapler hepatectomy

Definition and assessment: The entire BL from skin incision to skin closure is defined as “intraoperative BL”. For the assessment of the exact amount of hemorrhage the volume of fluid in the suction container(s) that were used during the procedure are recorded in the case report form (CRF) by the end of the skin closure. Furthermore the numbers of all small and big surgical swabs are recorded. All swabs together with the container that catches dripping excess fluids underneath are weighed by the end of skin closure and the exact weight in grams is recorded in the CRF. The exact amount of rinse fluid in milliliters that was used during the procedure is recorded in the CRF.

The calculation of total BL is as follows: The suction container fluid volume (in milliliters) is added to the weight (in grams) of all surgical swabs and the drip catching container at the end of the skin closure (A). The amount of ascites that may be suctioned from the abdominal cavity initially is subtracted from that volume. The difference of the density of the rinse solution (isotonic sodium chloride solution) and blood is approximately 0.055 g/cm^3^. With regard to the exactness of the measurements this difference is considered clinically irrelevant.

The volume of the entire rinse fluid (in milliliters) that was used during the procedure is added to the known dry weight (in grams) of the respective number of surgical swabs that were used during the procedure and the known weight of the empty container they are weighed in (B).

The total BL is defined as “A” minus “B” in milliliters.

### Secondary endpoints

- Intraoperative BL per resection plane size (ml/cm^2^)
- Number of packed red blood cells (PRBC) transfused intraoperatively
- Postoperative ALT levels day 1 and 3 (and their relative delta)
- Postoperative total bilirubin levels day 1 and 3 (and their relative delta)
- Postoperative INR day 1 and 3 (and its relative delta)
- Number of PRBC transfused within the first three postoperative days
- General surgical complications, classified according to the “Clavien – Dindo” classification within the first 30 postoperative days
- Mortality during the first ninety postoperative days

### Tested method

All procedures are performed according to institutional standards for liver surgery. Depending on the planned procedure, an upper midline incision with or without right lateral extension (“reversed L – shape”) is performed in a regular dorsal position. After the hepatic lesion is confirmed to be treatable by resection, the liver is freed from its ligaments and mobilized. In the treatment group Classic PM is performed via silicon tube tourniquet of the portal triad just before the resection starts. If the parenchymal resection exceeds fifteen minutes, the tourniquet will be loosened for five minutes followed by intermittent PM with five minute intervals of ischemia and reperfusion until the parenchymal resection is finished. Liver resection itself is performed in the stapler technique as described elsewhere [14]. Briefly, after identification of the resection plane, demarcation of the resection line by ligation of the specific hepatic arterial and hepatic vein branches, the parenchyma is stepwise fractured with a straight vascular clamp and dissected with an Endo GIA vascular stapler (Medtronic, Endo GIA™ Universal Roticulator 60–2.5 or Endo GIA™ Curved Tip 60 mm Articulating Vascular/Medium Tri-Staple™) in an alternating manner. After removal of the resected specimen, pressure with a hot, wet surgical swab is applied to the resection plane and PM is terminated immediately thereafter by loosening of the tourniquet. In all cases the central venous pressure will be kept at 5 mmHg or below by the anaesthesiologists according to routine liver surgery standards in our institution.

### Risks of the tested method

Liver resection naturally carries the risk of bleeding, independent of the resection technique used. After PM was first described as a means of haemorrhage control in liver trauma [15], it has been applied by liver surgeons to limit blood loss during elective liver resections. Potential harmful effects of PM have been widely discussed over decades. Based on the work of Huguet from 1994 it is widely accepted that warm ischemia due to PM is tolerated by healthy, non-cirrhotic livers for up to sixty minutes [16]. The parenchymal damage caused by ischemia reperfusion is commonly monitored by measurement of perioperative Liver transaminase levels. A meta-analysis on the use of PM in 2008 showed no statistical difference in Transaminase levels when PM was compared to hepatic resection without vascular occlusion [12]. Liver cirrhosis is prevalent in many cases of hepatic resections, especially for HCC. Based on experimental [17] and clinical [18–20] data, cirrhotic livers are more sensitive to ischemic damages including inflow occlusion. This effect is believed to be strongly dependent on the duration of the ischemic period. Accordingly, a clinical trial from 2004 found an advantage for intermittent portal clamping compared to continuous clamping in cirrhotic patients [21]. In this trial short ischemic periods of five minutes, followed by five minutes of reperfusion repetitively, were found to decrease the ischemia reperfusion damage in cirrhotic livers indicated by blood tests.

PM has been proven to be applied safely for up to sixty minutes in non-cirrhotic livers. By intermittent inflow occlusion the duration of resections under ischemia could be extended to up to 120 min [22]. To prevent potential harm in single cases with prolonged duration of parenchymal resection, after fifteen minutes the tourniquet will be loosened for five minutes. In the following intermittent PM with five-minute intervals of ischemia and reperfusion will be applied until the parenchymal resection is finished. Considering the average duration of parenchymal resection in this trial to be less than ten minutes in total, no harmful effects are expected to be caused by PM in the treatment group.

### Inclusion criteria

- Patients undergoing open left or right hepatectomy according to the Brisbane classification for benign or malignant diseases)
- Age of eighteen years or older
- Written informed consent for participation in the trial
- Absence of a mental state preventing the subject to understand the study related information

### Exclusion criteria

- Situation post interventional or operative portal vein embolization or ligation
- Anatomical alterations which render study treatment impossible (e.g. a biliodigestive anastomosis, an extraanatomic hepatic artery bypass, and/or any other circumstance which renders PM or stapler resection impossible)
- Simultaneous resection of other organs (excluding the gall bladder and the common bile duct)
- Liver cirrhosis exceeding Child – Pugh score A

### Patient selection

**Fig. 1:**
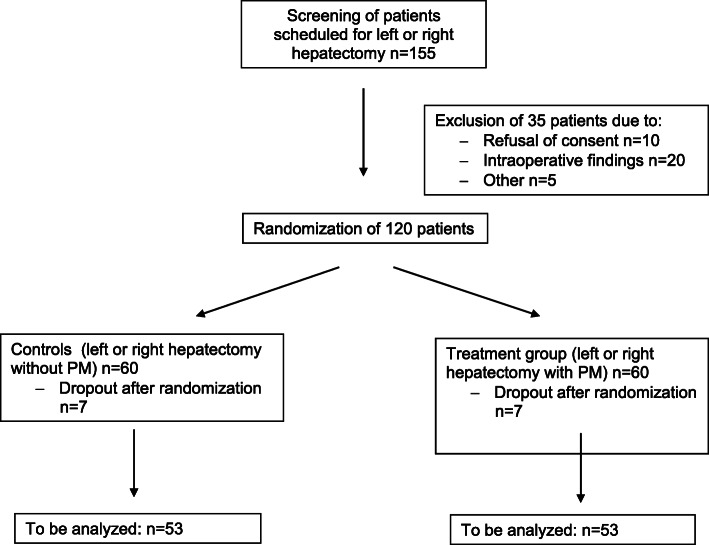
Trial Flowchart according to CONSORT

All patients who are scheduled for open left or right hepatectomies are screened for participation in the trial by a member of the Clinical Trial Center (KSC) of the Department of Surgery, University Hospital Heidelberg. Written informed consent is obtained from every subject on the day before surgery the latest. Due to expected drop outs because of intraoperative exclusion of screened patients, approximately 155 patients will be screened to include 106 patients in the final analysis. Figure 1 gives an overview of the patients' course through the trial. According to the above-mentioned inclusion and exclusion criteria subjects are randomized equally to either of the two groups intraoperatively:

- Controls – liver resection applying standard stapler parenchymal resection technique without hepatic inflow control as described previously [14]
- Treatment group – liver resection applying standard stapler parenchymal resection technique with continuous PM (intermittent PM after fifteen minutes of parenchymal resection)

### Randomization

#### Randomization process

The randomization will facilitate numbered, opaque envelopes, derived from a computer-generated random list by a member of our institution who is not involved in the trial otherwise. After the surgeon has performed the laparotomy and verified that the resection can be performed as planned without any impediments to the trial treatments, the KSC will be informed. The allocation to the treatment or control group in a 1:1 ratio will be done by a member of the KSC by opening the respective envelope and immediate informing of the operating team about the result. The result of the randomization will be recorded in the CRF. The stratification used for randomization is the type of resection: left / right hepatectomy.

### Data collection

Preoperatively, the patients´ general demographic and medical data will be taken from the existing clinical database (summarized in Table 1). According to the study endpoints the following data will be recorded intraoperatively in the CRF:

- Intraoperative BL (as defined and described under 2.3.1)
- Resection plane surface in cm^2^ (obtained by printing a standard DIN A 4, 1 × 1 cm checkered paper sheet with the resected Liver; boxes on the edges of the print < 50% coverage are neglected, boxes ≥50% coverage are counted as one cm^2^)
- Duration of parenchymal resection in minutes
- Duration of PM in minutes (if intermittent PM is applied, number and duration of occlusion periods)
- Type of resection (left or right hepatectomy according to the Brisbane 2000 terminology)
- Time from skin incision to completion of skin closure in minutes
- Number of PRBC transfused intraoperatively
- Protocol violations

**Table 1:** Preoperatively recorded baseline data

General	Age / gender / ASA status
General medical	Previous abdominal surgery/previous hepatic surgery/chronic viral hepatitis/cirrhosis/blood coagulation disorders
Indication for resection	Benign/primary malignant/secondary malignant / other
Preoperative blood results	ALT/AST/total bilirubin/GGT/AP/INR/total albumin

ASA: American Society of Anaesthesiologists; ALT: alanine aminotransferase; AST: aspartate aminotransferase; GGT: gamma glutamyl transferase; AP: alkaline phosphatase; INR: international normalized ratio

To analyse inter-group comparability of differences in the intraoperative BL, the central venous pressure (in mmHg) during the resection is recorded.

Postoperatively, the following data will be recorded or taken from the clinical database:

- Plasma ALT levels in U/L on postoperative day 1 and 3 (and their relative delta)
- Plasma Total bilirubin levels in mg/dl on postoperative day 1 and 3 (and their relative delta)
- INR on postoperative day 1 and 3 (and its relative delta)
- Numbers of transfused PRBCs up to postoperative day 3
- Incidence and type of a biliary leakage according to the Koch classification within the first 30 postoperative days [23]
- General surgical complications, classified according to the “Clavien-Dindo” classification within the first 30 postoperative days [24]
- Mortality up to day 90
- Duration of hospital stay

All generated data in the trial will be fully accessible to the principal investigator PH exclusively.

### Trial duration

Based on the sample size calculation (2.4.1), the evaluation of the last patient will be completed (last patient out) 31 months after the start of screening for eligible patients (first patient in). Another three months later the results of the data analysis will be available. The individual duration of the trial for each patient is 91 days (randomization on the day of surgery, follow up 90 days postoperatively). Table 2 gives an overview of the trial schedule according to the SPIRIT statement [25].

**Table 2:** Trial visits schedule

Trial visits					
Trial activity/examination	Preoperatively	Day of surgery	POD 1 and 3	POD 30	POD 90
Screening, informed consent, preoperative baseline data (Table 1)	X				
Type of resection (left/right hepatectomy)/BL/resection plane surface in cm^2^/number of transfused PRBC/duration of parenchymal resection/duration of PM/central venous pressure during resection/time from skin incision to completion of skin closure/protocol violations		X			
ALT/AST/total bilirubin/GGT/AP/INR/total albumin/numbers of transfused PRBCs/incidence of wound healing disorders/incidence and type of biliary leakage			X		
Incidence and type of wound healing disorders/incidence and type of biliary leakage/duration of hospital stay/mortality				X	
AE/SAE		X	X	X	
Mortality					X

*POD* Postoperative day, *BL* blood loss, *PRBC* packed red blood cells, *PM* Pringle Maneuver, *ALT* alanine aminotransferase, *INR* international normalized ratio, *AE* adverse event, *SAE* serious adverse event

### Termination of the trial

#### Individual criteria

If a patient withdraws his consent to participate in the trial, no further data collection is done and all of his data will be deleted upon demand. The patient will be asked for the allowance to analyse the data that was collected before the withdrawal of consent.

#### Termination of the entire trial

If new findings during the course of the trial reveal any safety concerns regarding the investigated method, all trial activity will be interrupted. If substantial safety concerns remain, the entire trial will be terminated.

Scheduled termination of the trial is when at least 53 patients in both trial arms have completed the follow up.

### Sample size

In our institution major liver resections are commonly performed without PM. Based on the intraoperative course, e.g. if extensive BL during parenchymal resection occurs, PM is performed according to the surgeon’s appraisal. In a retrospective analysis of 193 major liver resections that were performed in our institution from January 2011 until February 2014, we found a reduction of the mean intraoperative BL of 27.4% (647 (+/− 612) vs. 891 (+/− 783) ml) in the 62 cases with PM. Since PM was only used when an extensive, uncommon BL was detected, we expect the effect of PM to be substantially higher among standard major liver resections in general. Even though it is a generally accepted fact that blood loss and PRBC transfusions during liver surgery negatively affect patients’ outcome, it is uncertain if the effect is straight proportional. In a recent work Wehry et al. showed that the average amount of blood loss in 186 patients undergoing liver resection was reduced by approximately 400 ml if transfusions were not required [10]. In this context it appears reasonable that this interventional trial should be designed powerful enough to detect a reduction of blood loss of at least 400 ml. The sample size calculation for this trial is therefore based on the assumption that a reduction of the intraoperative BL has to be at least 50% (a reduction of approximately 450 ml) to be clinically relevant. Therefore, 53 patients have to be analyzed in each group to detect a 50% reduction of BL at a standard deviation of 94.1%, an alpha of 5% and an 80% power level. Assuming a realistic dropout rate due to intraoperative findings, contraindications for major liver resection and other reasons 155 patients are planned to be screened and asked for participation in the trial.

### Statistical methods

The non-parametric Mann-Whitney u test will be used to compare the intraoperative BL values between both treatment groups.

The Fisher’s exact test will be performed to compare the rates of 30-day mortality, bile leakage, intraoperative transfusion requirements, and blood transfusion requirements within the first three postoperative days between both treatment groups.

Depending on the distribution of the quantitative parameters intraoperative BL per resection plane size, duration of surgical procedure, and duration of hospital stay the T-test in case of a normal distributed parameter or the non-parametric Mann-Whitney-U-Test will be used to compare both treatment groups.

The postoperative ALT levels, total bilirubin levels, and INR at day 1 and at day 3 will be compared between both treatment groups using the T-test or the Mann-Whitney-U-Test. The Bonferroni-Holm method will be used to adjust the *p*-values.

Two-sided p-values < 0.05 will be considered as statistically significant.

### Safety/adverse events

#### Specification of adverse−/serious adverse events

“Adverse event” (AE) covers any sign, symptom, and syndrome, that appears in a subject during the follow up of the clinical trial and that may impair the well-being of the subject.

AEs will be recorded on a special AE form in the CRF. The following information will be recorded:

- Date of appearance
- Description of symptom
- Duration of AE
- Treatment
- Severity code: mild, moderate or severe
- Possible Relationship to trial treatment
- Outcome

Symptoms that are commonly seen after major liver resections including postoperative pain, nausea and vomiting, delay of bowel movement, fatigue and lack of appetite will not be recorded as AE. AEs will be reported to the principle investigator in regular intervals throughout the study.

“Serious Adverse Event” (SAE) is **any** adverse event that occurs at any time during the follow up period, that results in death, is immediately life threatening, requires hospital admission, results in persistent or significant disability or incapacity or results in reoperation due to any reason. SAEs will be documented on a special SAE formula in the CRF and will be reported to the principle investigator within 24 h. The following information will be recorded:

- Date of appearance
- Description of symptom
- Duration of SAE
- Treatment
- Severity code: mild, moderate or severe
- Possible relationship to trial treatment
- Outcome

SAEs which meet one of definitions of the secondary endpoints are treated as SAEs regarding to documentation but have not to be reported to the sponsor/principle investigator within 24 h. They will be reported to the principle investigator in regular intervals throughout the study.

## Discussion

PriMal StHep is the first randomized clinical trial to test the combination of PM and stapler resection technique as a means to reduce intraoperative blood loss in hemi-hepatectomies. The ongoing debate about potential adverse effects of PM in major liver resection is mainly attributed to extended durations of parenchymal dissection. As stapler hepatectomy has been proven to offer safe time- and cost-effective parenchymal dissection, the addition of PM to further reduce the intraoperative blood loss appears promising.

### Trail status

The PriMaL StHep trial has been prospectively registered to the German Clinical Trial Registry (WHO ID: DRKS00010427) on April 21st, 2016.

The current protocol version is V. 1.5 from August 8th, 2017. The PriMaL StHep trial is recruiting since June 2016. Recruitment will be completed approximately in October 2018.

## Data Availability

The results of the trial will be made publicly available via publishing in a peer reviewed journal.

## Abbreviations

AE: Adverse Event
ALT: Alanine Aminotransferase
AP: Alkaline Phosphatase
ASA: American Society of Anaesthesiologists
AST: Aspartate Aminotransferase
BDSG: Bundesdatenschutzgesetz
BL: Blood Loss
CVP: central Central venous pressure
GGT: Gamma Glutamyl Transferase
HCC: Hepatocellular Carcinoma
HMGB 1: High Mobility Group Box 1
INR: International Normalized Ratio
IPM: Intermittent Pringle Maneuver
KSC: Clinical Trial Center
PM: Pringle Maneuver
POD: Postoperative day
PRBC: packed red blood cells
RCT: Randomized Controlled Trial
SAE: Serious Adverse Event
THVE: Total Hepatic Vascular Exclusion
